# Optimization of the Electronic Health Record for Research

**DOI:** 10.1097/AS9.0000000000000297

**Published:** 2023-06-13

**Authors:** Jacqueline A. Murtha, Dawda Jawara, Luke M. Funk

**Affiliations:** From the * Department of Surgery, University of Wisconsin-Madison, Madison, WI; † Department of Surgery, William S. Middleton Memorial Veterans Administration Hospital, Madison, WI.

## Abstract

Mini Abstract This surgical perspective highlights the current limitations of utilizing the electronic health record for research and discusses directions for future optimization.

The electronic health record (EHR) comprises a longitudinal collection of data that builds as patients interface with the medical system. EHRs initially emerged in the 1980s to improve the quality of care and optimize healthcare billing. The role of EHRs grew substantially following increased incentivization for their use as part of the Health Information Technology for Economic and Clinical Health Act, which was passed by the US Congress in 2009.^1^ In addition to benefits that the EHR provides for patient care and billing, it has numerous attributes that make it useful for research. Namely, it serves as a centralized location for longitudinal medical data that can be analyzed for basic science, translational, and clinical research questions.

## CURRENT LIMITATIONS OF USING THE EHR FOR RESEARCH

Since the EHR was not designed for research and its data are either autopopulated (eg, laboratory results) or manually inputted by non-research staff for clinical and billing purposes, there are numerous barriers to conducting EHR-based research. These include concerns regarding data quality, lack of integration across healthcare and EHR systems, and limited types of data available for extraction.

### Data Quality

**FIGURE 1.**
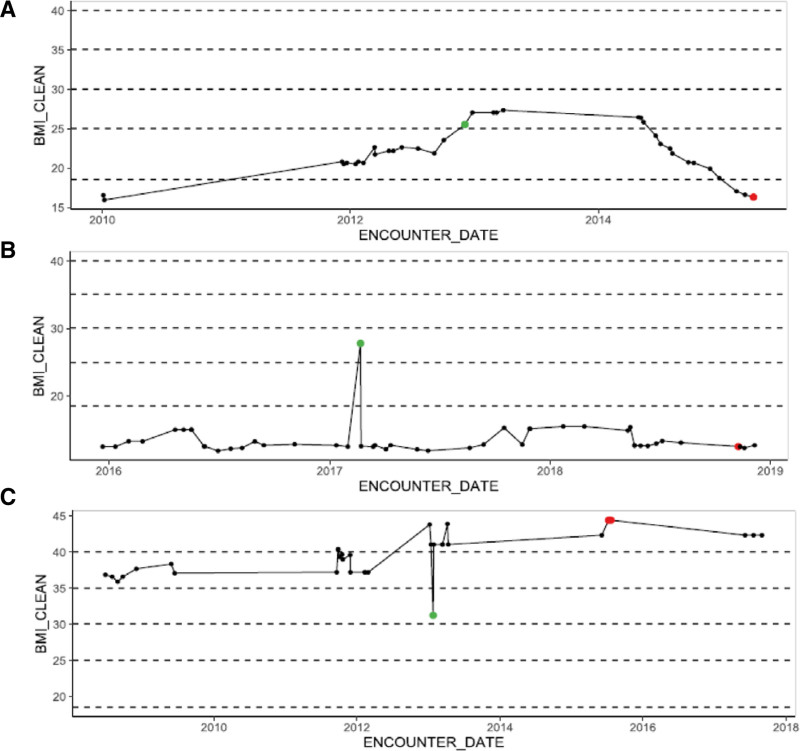
Body mass index trend examples. Body mass index (BMI) trends from three patients in University of Wisconsin electronic health record. A, Realistic decrease in BMI given number of corroborating data points. B and C, Examples felt to be the BMI data errors given abrupt spikes.

Given the lack of requirements for routine monitoring, auditing, and verification of EHR data^2^ and the lack of a standardized approach to “data cleaning” (i.e., identifying and correcting data entry errors through removal, replacement, or modification), the quality of EHR data can be problematic when used for research purposes. Additionally, many datasets used for research, such as the American College of Surgeons National Surgical Quality Improvement Program, extract data from the EHR. In our EHR-based research projects, we routinely encounter erroneous laboratory (eg, diluted samples from improper collection or laboratory errors such as hemolyzed samples) and vital sign (eg, falsely low blood pressures confirmed to be normal on recheck) data while performing analyses. These values are often not expunged from the EHR and continue to propagate forward. Despite using an algorithm to remove implausible height and weight data in the EHR, our group found that approximately 5% of weights were erroneous upon chart review. **Figure 1** displays body mass index (BMI) trends for three patients in our EHR dataset. Two patients likely have BMI data entry errors.

### Lack of Integration and Standardization

Lack of EHR integration across healthcare systems prevents automatic record linking, which results in an incomplete representation of a patient’s medical history. There are over 20 EHR vendors in the United States, and the average healthcare system is associated with 18 different EHR vendors when considering inpatient and outpatient clinics and affiliated providers.^3^ Most of these EHRs lack interoperability, or the ability to communicate with each other. For example, a patient who receives care in a Cerner-based healthcare system would not have records that could be shared with an Epic-based healthcare system. Similarly, while the “Care Everywhere” feature within Epic allows records from other healthcare systems to be viewed, these records are not part of the main EHR and must be searched separately (eg, an echocardiogram performed at another institution will not be visible within the procedures section).

The lack of a common data model and data definitions also makes it difficult to compare EHR analyses.^4^ For instance, laboratory variables may have different units or be recorded in variable formats and locations. This complicates data analysis even within a single healthcare network.

### Limited Types of Data

Despite the wide range of data stored within EHRs, all data are not available for extraction. Information regarding the social history is often either absent or outdated, and free-text data such as sociodemographic or lifestyle habits are not available. Variables such as education, income, living environment, and diet may be more informative than the social history recorded in the EHR (employment, smoking and alcohol use, etc.), but they are not readily available. Furthermore, data is spread between structured (eg, ICD and CPT codes) and unstructured (eg, social history free text) formats complicating extraction. One option for mitigating this weakness is with natural language processing, which is a form of artificial intelligence that can extract and label the desired data from free text using techniques such as key word recognition and entity linking.^4^ For instance, natural language processing can be used to extract pathology specimen findings such as margin, invasion, and tumor characteristics from free-text reports.

## FUTURE DIRECTIONS FOR EHR-BASED RESEARCH

### Methods to Improve Data Quality

Rule-based methods are the most common tool to ensure data quality, although none are widely implemented across different systems. For example, our research group uses a methodology proposed by Cheng et al. for “cleaning” of BMI data.^5^ In an effort to systematically improve data quality, researchers at another tertiary care center developed more than 60,000 rules that flagged over 800,000 data discrepancies among 1.46 million patient charts.^6^ Examples of these data discrepancies included biologically implausible temperature values and dates of birth and prostate cancer in a patient with female sex. While having these types of rules may improve data quality, a hybrid approach involving both automated checks and human oversight may be needed to address the discrepancies.^6^ Rule-based methods are also less reliable at the extremes. For instance, a rule-based algorithm for height, weight, and BMI data quality assessment was less likely to correctly include or exclude weight and BMI data for individuals with underweight and obesity relative to those in normal weight ranges.^2^

The future of EHR research will encompass optimization of data entry and abstraction, along with increased utilization of machine learning approaches for big data analysis. To address data entry, some have proposed data validation at the time of entry (eg, human checks of laboratory and vital sign values prior to publication in EHR) and further incorporation of speech recognition technology, which would decrease typing/clicking and human error.^1,2^ These proposals are not without weaknesses—data validation at the time of entry is time-consuming and resource intense, while speech technology can result in inaccurate transcriptions. To optimize data abstraction, EHR queries will need to be standardized and utilize natural language processing along with improved data processing techniques to remove erroneous data entries. There is also a push for the “fundamental redesign of the EHR to improve data entry and retrieval” with an emphasis on “goal-oriented functionality.”^1^

### Real-time Analysis

Due to the increase in computing power and available data for analysis, machine learning approaches ranging from regularized regression to gradient-boosted decision trees to neural networks have been increasing in popularity over the past decade. They allow for the simultaneous analysis of many variables to explore associations between variables. These algorithms can also be incorporated into the EHR to allow for real-time prediction that continues to learn. Aside from prediction, machine learning approaches have been used in radiology to generate imaging reports. Ultimately, as data are standardized and natural language processing improves, we can transition to a “smart EHR” that can process contextual information in real-time and serve an active role in the provision of clinical care. The incorporation of predictive algorithms into the EHR is not without risk as they are only as “smart” and “bias-free” as the developers and data on which they are trained.

### New EHR-based Research Networks

The realization that large amounts of accessible, quality data are needed to advance medical research has led to the development of several large research networks over the last decade. First, the National Institutes of Health has created the *All of Us* Research Program, which has enrolled nearly 330,000 individuals. Patients in this dataset have completed an enrollment survey, which is linked to their EHR data and is subsequently standardized via the Observational Medical Outcomes Partnership data model.^7^ Second, the Veteran Affairs Million Veterans Program (MVP) is an ongoing observational cohort of Veterans that includes survey information, biospecimen data and access to EHR data for more than 800,000 Veterans.^8^ Both the *All of Us* and MVP datasets also contain genomics data on thousands of patients. Epic Cosmos is another example of an EHR company engaging in dissemination of data with goal to provide rapid access and collaborative research. Cosmos allows for data mining of 120 million patient records nationwide to produce reports.^9^ Finally, the National Patient-Centered Clinical Research Network (PCORnet) is a research network that uses a common data platform alongside the existing EHR. It allows for observational and interventional comparative effectiveness research, and is supported by institutions and health care systems that represent more than 60 million patients in the United States. PCORnet relies on rule-based data cleaning that is done in an iterative fashion to allow for continued expansion and improvement in data quality.^10^ Both Cosmos and PCORNet require organizations to pay to utilize the data. These datasets may help address shortcomings of EHR data and spur the development of improved algorithms for data quality screening that can subsequently be implemented by institutions.

There are also emerging areas of EHR use for research including use of EHRs as registries for randomized control trials and patient recruitment, comparative effectiveness trials, and active surveillance. For example, Sentinel with the Food and Drug Administration proactively monitors medical product safety post-marketing using EHR data.^4^

In conclusion, widespread adoption of the EHR has created rich research opportunities due to a dramatic increase in the amount and accessibility of patient data. However, there are ongoing concerns regarding data quality, lack of system integration creating an incomplete picture, and difficulty extracting subsets of data, such as free-text data. Large, EHR-based datasets have emerged to address these shortcomings and facilitate EHR-based research dissemination. These new networks combined with improvements in computing power will usher in a new era of “big data” research to improve healthcare and patient outcomes.

## ACKNOWLEDGMENT

J.A.M., D.J., and L.M.F. participated in writing of the paper.
